# Healthcare provision for insect venom allergy patients during the COVID-19 pandemic

**DOI:** 10.1007/s40629-020-00157-z

**Published:** 2020-12-08

**Authors:** Margitta Worm, Barbara Ballmer-Weber, Randolf Brehler, Mandy Cuevas, Anna Gschwend, Karin Hartmann, Thomas Hawranek, Wolfram Hötzenecker, Bernhard Homey, Thilo Jakob, Natalija Novak, Julia Pickert, Joachim Saloga, Knut Schäkel, Axel Trautmann, Regina Treudler, Bettina Wedi, Gunter Sturm, Franziska Rueff

**Affiliations:** 1 Department of Dermatology, Venereology and Allergology, Charité—University Hospital Berlin, Berlin, Germany; 2 Department of Dermatology, Venereology and Allergology, Cantonal Hospital St. Gallen, St. Gallen, Switzerland; 3 Department of Dermatology, University Hospital Münster, Münster, Germany; 4 Department and Outpatient Clinic for Otorhinolaryngology, Carl Gustav Carus University Hospital Dresden, Dresden, Germany; 5 Outpatient Clinic, University Department of Rheumatology, Immunology and Allergology, University Hospital Bern, Bern, Switzerland; 6 Allergology and Dermatology, University Hospital Basel, Basel, Switzerland; 7 University Department for Pediatric and Adolescent Medicine, Paracelsus Private Medical University, Salzburg, Austria; 8 Department of Dermatology and Venereology, Kepler University Hospital GmbH, Linz, Austria; 9 Department of Dermatology, University Hospital Düsseldorf, Düsseldorf, Germany; 10 Deptartment of Dermatology and Allergy, University Medical Center Gießen (UKGM), Justus Liebig University Gießen, Gießen, Germany; 11 Department and Outpatient Clinic for Dermatology and Allergology, University Hospital Bonn, Bonn, Germany; 12 Department of Dermatology and Allergology, University Hospital Gießen and Marburg GmbH, Philipps University Marburg, Marburg, Germany; 13 Department and Outpatient Clinic for Dermatology, Johannes Gutenberg University Mainz, Mainz, Germany; 14 Department of Dermatology, University Hospital Heidelberg, Heidelberg, Germany; 15 Department and Outpatient Clinic for Dermatology, Venereology and Allergology, University Hospital Würzburg, Würzburg, Germany; 16 Department and Outpatient Clinic for Dermatology, Venereology and Allergology, University Hospital Leipzig, Leipzig, Germany; 17 Department of Dermatology, Allergology and Venereology, Hannover Medical University, Hannover, Germany; 18 University Department of Dermatology and Venereology, Medical University of Graz, Graz, Austria; 19 Department and Outpatient Clinic for Dermatology and Allergology, University Hospital Munich, Munich, Germany; 20 Division of Allergy and Immunology, Department of Dermatology, Venereology, and Allergology, Charité—Universitätsmedizin Berlin, Charitéplatz 1, 10117 Berlin, Germany

**Keywords:** Venom, Allergy, Immunotherapy, SARS-Co-2, Lockdown

## Abstract

The population prevalence of insect venom allergy ranges between 3–5%, and it can lead to potentially life-threatening allergic reactions. Patients who have experienced a systemic allergic reaction following an insect sting should be referred to an allergy specialist for diagnosis and treatment. Due to the widespread reduction in outpatient and inpatient care capacities in recent months as a result of the COVID-19 pandemic, the various allergy specialized centers in Germany, Austria, and Switzerland have taken different measures to ensure that patients with insect venom allergy will continue to receive optimal allergy care. A recent data analysis from the various centers revealed that there has been a major reduction in newly initiated insect venom immunotherapy (a 48.5% decline from March–June 2019 compared to March–June 2020: data from various centers in Germany, Austria, and Switzerland). The present article proposes defined organizational measures (e.g., telephone and video appointments, rearranging waiting areas and implementing hygiene measures and social distancing rules at stable patient numbers) and medical measures (collaboration with practice-based physicians with regard to primary diagnostics, rapid COVID-19 testing, continuing already-initiated insect venom immunotherapy in the outpatient setting by making use of the maximal permitted injection intervals, prompt initiation of insect venom immunotherapy during the summer season, and, where necessary, using outpatient regimens particularly out of season) for the care of insect venom allergy patients during the COVID-19 pandemic.

At a population prevalence of 3–5%, insect venom allergy is common and can potentially trigger life-threatening allergic reactions [1]. Therefore, patients who have experienced a systemic allergic reaction to an insect sting should be referred to an allergy specialist for diagnosis and treatment. In addition to patient history taking, where the symptoms and concomitant circumstances of the reaction are recorded, the standard procedure includes titrated skin prick testing and, if necessary, intracutaneous testing and/or determination of specific immunoglobulin (Ig)-E antibodies to insect venom and, where appropriate, their components to identify immediate-type allergy (Fig. 1). For a better risk assessment, especially after the onset of severe reactions, the determination of basal serum tryptase is also recommended. If the above-mentioned findings are positive and the patient has a clear history of a systemic allergic reaction in the context of a venom sting, the initiation of allergen-specific immunotherapy with the relevant insect venom is recommended [2].

**Fig. 1:**
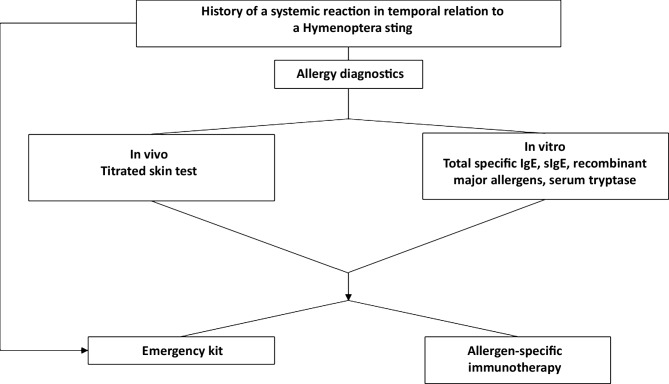
Diagnostic algorithm for insect venom allergy (from [2]). *IgE* immunoglobulin E, *sIgE* specific immunoglobulin E

The failure to initiate specific immunotherapy in at-risk patients in a timely manner, leads to an increase of their health risks and may result in an increased need of emergency care for insect sting reactions. Such situations should be avoided during possible healthcare shortage. The significance of the COVID-19 pandemic for allergology has recently been discussed in a number of position papers [3, 4]. Due to the widespread reduction in outpatient and inpatient care capacities in recent months as a result of the COVID-19 pandemic, the various allergy specialists from Germany, Austria, and Switzerland have taken different measures to ensure that patients with insect venom allergy continue to receive optimal allergy care. However, overall, there has been a large reduction in newly initiated insect venom immunotherapy (Table 1) during the lock down. A survey among large allergy centers with regard to newly initiated venom immunotherapy (VIT) revealed an almost 50% reduction for the months March–June 2020 compared to the similar period in 2019 (Fig. 2). This decline was related to reduced hospital capacities, but also the fact that patients considered to visit a physician or a hospital as a high-risk due to the COVID-19 pandemic.

**Table 1:** Overview of the number of VIT initiated in the period March–June 2019 and 2020 at a number of different centers

Centers	Initiated VIT March–June 2019	Initiated VIT March–June 2020
Allergology and Dermatology, University Hospital Basel, Switzerland	Not specified	Not specified
Department of Dermatology, Venereology and Allergology, Charité—University Hospital Berlin, Germany	28	9
Outpatient Department, University Department of Rheumatology, Immunology and Allergology, University Hospital, Switzerland	~30	12
Department and Outpatient Clinic for Dermatology and Allergology, University Hospital Bonn, Germany	28	21
ENT Department and Outpatient Clinic, Carl Gustav Carus University Hospital Dresden, Germany	23	25
Department of Dermatology, University Hospital Düsseldorf, Germany	Not specified	Not specified
Department of Dermatology and Allergology, University Hospital Gießen and Marburg, Germany	76 (36 G, 40 M)	42 (33 G, 9 M)
University Department of Dermatology and Venereology, Medical University of Graz, Austria	50	2
Department of Dermatology, Allergology and Venereology, Hannover Medical University, Hannover, Germany	89	18
Department of Dermatology, University Hospital Heidelberg, Germany	15	17
Department and Outpatient Clinic for Dermatology, Venereology and Allergology, University Hospital Leipzig, Germany	35	33
Department of Dermatology and Venereology, Kepler University Hospital, Linz, Austria	31	16
Department and Outpatient Clinic for Dermatology, Johannes Gutenberg University Mainz, Germany	Not specified	Not specified
Department and Outpatient Clinic for Dermatology and Allergology, University of Munich, Germany	87	53
Department of Dermatology, University Hospital Münster, Germany	61	40
University Department for Pediatric and Adolescent Medicine, Paracelsus Private Medical University, Salzburg, Austria	29	17
Department of Dermatology, Venereology and Allergology, Cantonal Hospital St. Gallen, Switzerland	20	5
Department and Outpatient Clinic for Dermatology, Venereology and Allergology, University Hospital Würzburg, Germany	Not specified	Not specified

*VIT* venom immuntherapy, *G* Gießen, *M* Marburg

**Fig. 2:**
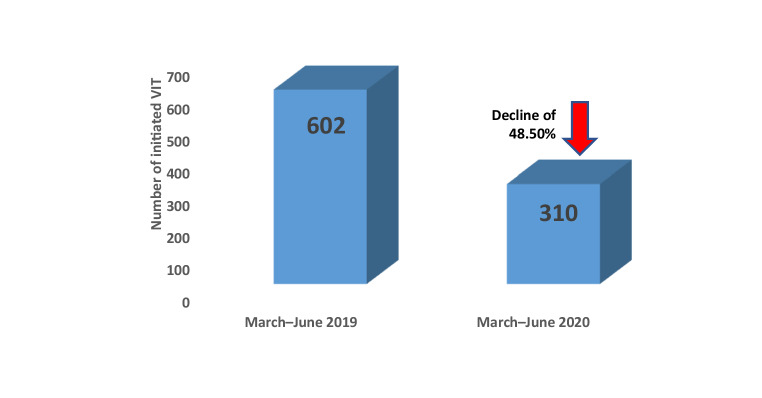
Number of initiated VIT (total from 14 centers in Germany, Austria, and Switzerland) between March and June in 2019 compared to 2020. *VIT* venom immunotherapy

Thus, the authors propose measures to ensure allergy care for insect venom-allergic individuals during times of emergency regulations in the healthcare system, such as during the COVID-19 pandemic (Table 2).

**Table 2:** Recommended measures for the care of insect venom allergy sufferers during the COVID-19 pandemic

Increased use of telephone and video appointments
Rearranging waiting areas and implementing hygiene measures and social distancing rules at stable patient numbers
Collaboration with practice-based physicians with regard to primary diagnostics (determination of bee/wasp sIgE)
Rapid COVID-19 testing (preadmission or on admission)
Unrestricted outpatient continuation of already-initiated insect venom immunotherapy (except in patients suffering from COVID-19 themselves) by making use of the permitted injection intervals
Prompt new initiation of insect venom immunotherapy during the season; if necessary, use of outpatient protocols, especially out of season
Explicitly addressing the COVID-19 situation with patients (either personally or in the scheduling letter)
In the case of shortages, triage according to the severity of the sting reaction
Adapting departmental organization, e.g., collaboration with other departments, extended outpatient clinic times, up-titration at weekends

*sIgE* specific Immunoglobin E

## Continuation of already-initiated insect venom immunotherapy

Allergen-specific immunotherapy with insect venom that has already been initiated should be continued as consistently as possible, despite eventual limitations in medical resources, by making use of the permissible length of intervals (see also [3]). Interrupting specific immunotherapy can cause a loss of protection and leads to unnecessary expense at a later point as a result of having to re-start therapy if the treatment interval has been exceeded. If the patient has COVID-19 themselves, a pause in treatment is recommended until recovery. Following recovery, the dose should be re-up-titrated (if still within the permitted interval) or allergen-specific immunotherapy newly initiated if necessary. In some cases, it may be beneficial to contact the patient by telephone or telehealth appointment prior to their personal visit for the immunotherapy injection in order to rule out current contraindications to the injection, thereby potentially saving the patient an unnecessary visit.

## New initiation of insect venom immunotherapy

It is possible to postpone the new initiation of insect venom immunotherapy out of season, assuming the time window is taken into account (see also [3]). Postponing initiation therapy during the summer season should be avoided, in order that the patient is not exposed to the risk of a repeat severe reaction to an accidental sting. Treatment initiation should preferably be performed as ultra-rush therapy under medical supervision. One- to five-day protocols have proven successful to this end [5, 6]. They have the advantage that the maximum dose is achieved after a short initiation treatment phase. Shortened outpatient up-titration protocols have also been investigated for vespid venom allergy patients and show good results in terms of safety [7]. However, they require a longer initiation phase (7 weeks), implying that such treatment protocols should be preferred out of the season.

In summary, the diagnostic work-up of insect venom allergy, including the patient history and skin testing, should be adapted to the prevailing conditions. Initiation of immunotherapy should continue to be started with ultra-rush protocols and, above all, not postponed during the summer season. During the out-of-season period and in case of shortages of inpatient resources, or in case of certain regional requirements, an up-titration can be performed in an outpatient setting. A shortened, 7‑week protocol for vespid venom allergy patients has been recently published [7]. Whenever possible, outpatient up-titration should be performed at a center experienced with this therapy and is able to provide emergency medical care.

## References

[1] Worm M, Moneret-Vautrin A, Scherer K, Lang R, Fernandez-Rivas M, Cardona V (2014). First European data from the network of severe allergic reactions (NORA). Allergy.

[2] Przybilla B, Ruëff F, Walker A, Räwer HC, Aberer W, Bauer CP (2011). Diagnose und Therapie der Bienen- und Wespengiftallergie. Allergo J.

[3] Klimek L, Pfaar O, Worm M, Bergmann KC, Bieber T, Buhl R (2020). Allergen-Immuntherapie in der Aktuellen Covid-19-Pandemie. Allergo J.

[4] Shaker MS, Wallace DV, Golden DBK, Oppenheimer J, Bernstein JA, Campbell RL (2020). Anaphylaxis-a 2020 practice parameter update, systematic review, and grading of recommendations, assessment, development and evaluation (GRADE) analysis. J Allergy Clin Immunol.

[5] Brehler R, Wolf H, Kütting B, Schnitker J, Luger T (2000). Safety of a two-day ultrarush insect venom immunotherapy protocol in comparison with protocols of longer duration and involving a larger number of injections. J Allergy Clin Immunol.

[6] Lee H, Roediger C, Bauer A, Zuberbier T, Worm M (2006). Prospective safety analysis of an ultrarush specific immunotherapy in adults with wasp venom allergy. Allergy.

[7] Schrautzer C, Arzt-Gradwohl L, Bokanovic D, Schwarz I, Čerpes U, Koch L (2020). A safe and efficient 7-week immunotherapy protocol with aluminum hydroxide adsorbed vespid venom. Allergy.

